# First person – Zariah Tolman

**DOI:** 10.1242/dmm.049639

**Published:** 2022-06-01

## Abstract

First Person is a series of interviews with the first authors of a selection of papers published in Disease Models & Mechanisms, helping early-career researchers promote themselves alongside their papers. Zariah Tolman is first author on ‘ *Elp1* is required for development of visceral sensory peripheral and central circuitry’, published in DMM. Zariah conducted the research described in this article while an undergraduate research assistant in Frances Lefcort's lab at Montana State University, Bozeman, MT, USA. She graduated with a Master's degree under the advice of Mark Schure at Montana State University, investigating the mechanisms of how youth develop emotion regulation strategies and how emotional regulation relates to wellbeing.


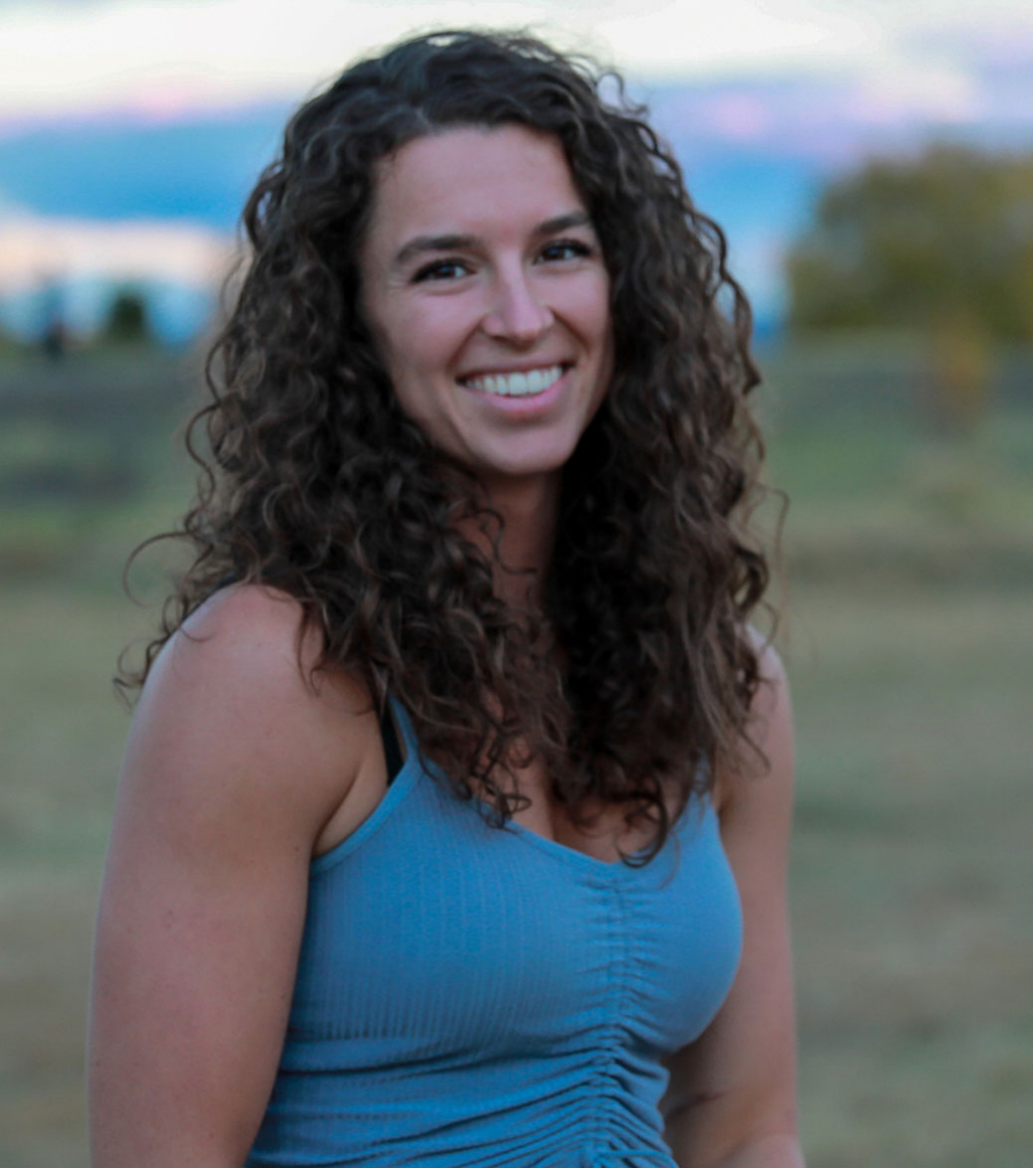


**Zariah Tolman**

**How would you explain the main findings of your paper to non-scientific family and friends?**

Our lab studies a disease called familial dysautonomia (FD). The disease affects people, but we study it in mice. The disease causes neurons that are important for your body to work to not develop properly. For example, there are neurons that sense your blood pressure at your heart and then send those signals up to your brain so that your brain can regulate your blood pressure effectively. We found that mice that have FD have fewer neurons than mice that are not sick, which means that the signals needed to regulate blood pressure are not making it up to the brain correctly.

“Our findings help explain why patients with FD struggle to regulate their blood pressure.”

**What are the potential implications of these results for your field of research?**

Our findings help explain why patients with FD struggle to regulate their blood pressure. As an example, a patient with FD heard her favourite food was in the next room, and the excitement was enough to spike her blood pressure so high she passed out. The results suggest that if those neurons can be rescued and prevented from dying, then patients might be able to better regulate their blood pressure and have improved quality of life.

**What are the main advantages and drawbacks of the model system you have used as it relates to the disease you are investigating?**

The mouse models our lab studies are conditional knockouts, so the gene that causes the disease (*Elp1*) is removed in specific tissues. Where the gene is removed depends on the promoter that was used. A complete knockout, where the gene is removed in all tissues, would be very advantageous because it more closely resembles the human patients; however, complete *Elp1* knockouts do not develop. Using four models that have the gene removed in different neuron populations, we have been able to piece together a picture of how FD affects each population.

Another advantage of mouse models is that we were able to generate mice that express green fluorescent protein (GFP) wherever the *Elp1* gene is removed. In the *Phox2b-cre* model, the neurons that sense the blood pressure fluoresce a beautiful green when visualized on a confocal microscope. We were able to see the stark contrast clearly and easily between the neurons of the sick mice and the healthy mice. Our lab was one of the first to use this model and technique to visualize the blood pressure sensory circuit, which is useful for a broader audience than just those interested in FD.

**Figure:**
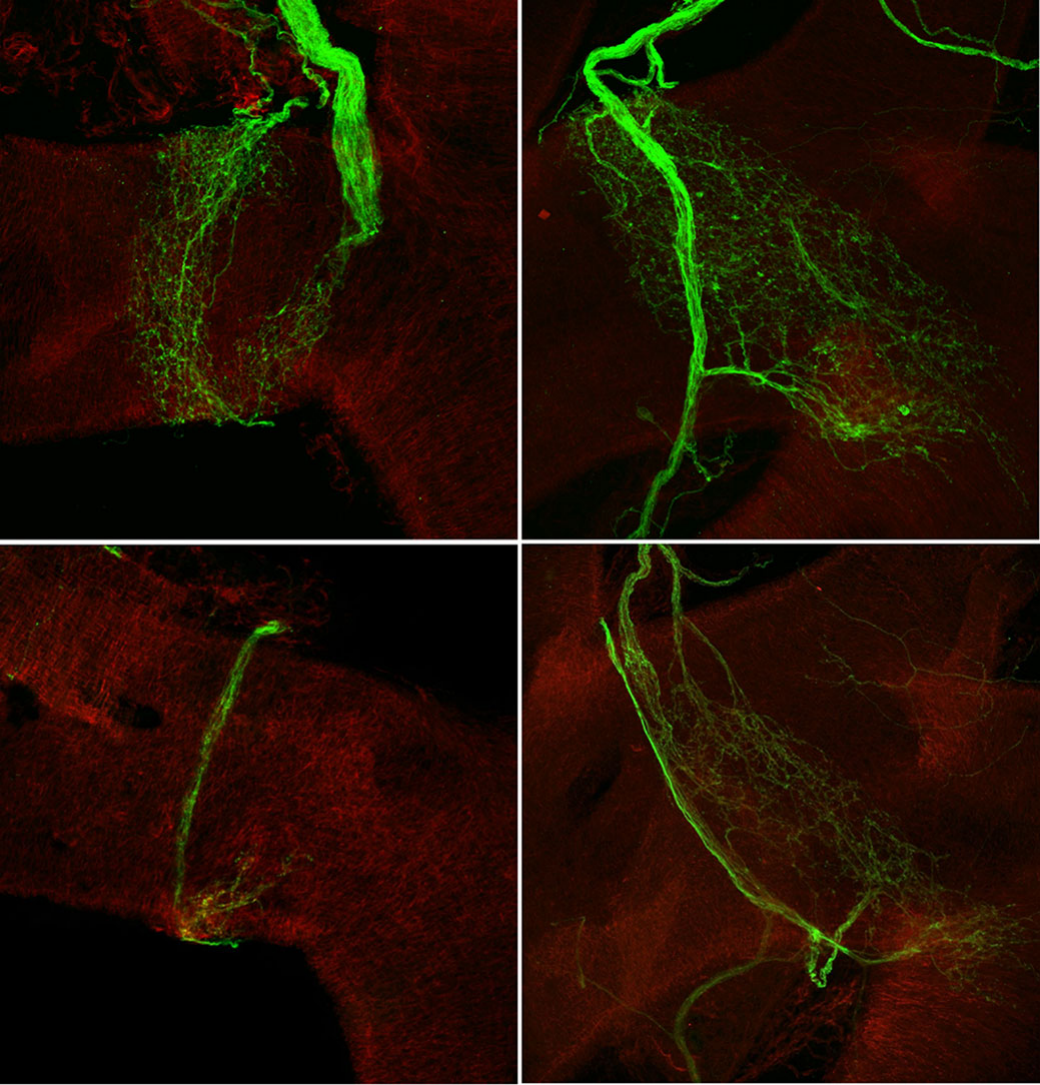
Neurons (green) that sense blood pressure near the heart are decreased in branching and density in FD mice (bottom panels) compared to control mice (top panels).

**What has surprised you the most while conducting your research?**

I was surprised at how relevant my research was to my other interests. Since FD is a very rare disease, I was not expecting my work to relate to my interest in mental health and youth development. However, the vagus nerve (what I studied in FD) plays an important role in trauma responses and how we experience our emotions. Understanding the nervous system within the context of FD has been very useful for my other research, where we are evaluating the effectiveness of an intervention that I developed to help youth learn to regulate their emotions.

**What's next for you?**

The research in this paper was conducted during my 3 years as an undergraduate. I graduate with a Master's degree in science in innovation and management this month (May 2022), and I will begin a PhD in developmental psychology at the University of California, Riverside this fall. I will continue my work with the nonprofit I founded, Positivity Outward, to empower youth with mentors for wellbeing. My research will investigate the mechanisms of how our program helps youth develop emotion regulation strategies.

**What is the one piece of advice you'd like to share with undergraduates looking to get into research?**

Research has many ups and downs, and that is okay. Sometimes things go well and you are very motivated, while sometimes nothing seems to work and you have low motivation. It's okay that you're going to be frustrated and burned out sometimes, so be persistent and curious about what you can learn even when you aren't motivated. Don't fight these natural ebbs and flows, because they're inevitable. Just keep moving, don't get stuck in the low parts, and definitely don't give up. Even if you decide that you do not want to be in research forever, you did not fail, and the experience will be extremely valuable for you.
